# HISTORI Experience – a qualitative study of HISTORI patients´ experiences with Semaglutide and placebo

**DOI:** 10.1192/j.eurpsy.2025.2252

**Published:** 2025-08-26

**Authors:** B. S. Rasmussen, N. Uhrenholt, C. Simonÿ, P. H. Gæde, B. Lundberg, S. T. Rønne, P. G. Uhrenholt, S. M. Arnfred

**Affiliations:** 1 Psychiatric Research Unit, Region Zealand.; 2 Psychiatry West, Region Zealand, Research Unit for Clinical Psychopharmacology, Slagelse; 3 Child and Adolescent Psychiatry Odense, Mental Health Services in the Region of Southern Denmark, Odense C.; 4 Department of Physiotherapy and Occupational Therapy, Næstved, Slagelse & Ringsted Hospital, Region Zealand, The Research and Implementation Unit PROgrez, Slagelse; 5 The Institute of Regional Health Research University of Southern Denmark, Odense; 6 Department of Cardiology and Endocrinology, Næstved, Slagelse & Ringsted Hospital, Slagelse; 7 Community Mental Health Services, Region Zealand, Vordingborg, Denmark

## Abstract

**Introduction:**

Qualitative studies of the connection between overweight/ obesity, medicine adherence and symptoms of psychosis are lacking. The project Home-based Intervention with Semaglutide Treatment of Neuroleptica-Related Prediabetes, HISTORI, was a blinded, randomized control trial where people with schizophrenia and prediabetes were randomized to either Semaglutide og placebo injections.

**Objectives:**

The research question in HISTORI Experience focused on the patients´ mindset while participating in HISTORI, their experiences and thoughts regarding weight and exercise, eating behaviors, psychotic symptoms and their interactions with HISTORI staff.

**Methods:**

19 qualitative, semi structured interviews were conducted. 11 Semaglutide interviews (6 female, 5 male) and 9 placebo interviews (5 female, 3 male). The interviews were analyzed with a reflexive thematic analysis (Braun & Clarke, 2022).

Patient and Public Involvement: One person with lived experience who could not be included in the HISTORI project, joined HISTORI Experience as a co-researcher. She was involved in creating a phenomenological interview guide. She was then introduced to the coding process and participated in the development of themes. A didactic relationship model was applied as an overall framework for the involvement (Hiim & Hippe, 2007). This model is learning-oriented and takes different factors that might influence the coorporation into account. Factors such as setting, learners´ abilities, learning process, content, goal and evaluation. To ensure a sustainable and active involvement the Involvement Matrix (Smits *et al*. Research Involvement and Engagement; 2020) was used both prospective and retrospective throughout the involvement process.

**Results:**

The results from both HISTORI and HISTORI Experience provides us with new knowledge that can be implementet in treatment with Semaglutide for patients with schizophrenia.

**Conclusions:**

Results and conclusion are expected in April 2025 and will be represented at the EPA conference.

**Disclosure of Interest:**

None Declared

